# Sintering of viscous droplets under surface tension

**DOI:** 10.1098/rspa.2015.0780

**Published:** 2016-04

**Authors:** Fabian B. Wadsworth, Jérémie Vasseur, Edward W. Llewellin, Jenny Schauroth, Katherine J. Dobson, Bettina Scheu, Donald B. Dingwell

**Affiliations:** 1 Department for Earth and Environmental Sciences, Ludwig-Maximilians-Universität, Theresienstr. 41, Munich 80333, Germany; 2 Department of Earth Sciences, Durham University, Science Labs, Durham DH1 3LE, UK

**Keywords:** relaxation, sphere packing, viscous sintering

## Abstract

We conduct experiments to investigate the sintering of high-viscosity liquid droplets. Free-standing cylinders of spherical glass beads are heated above their glass transition temperature, causing them to densify under surface tension. We determine the evolving volume of the bead pack at high spatial and temporal resolution. We use these data to test a range of existing models. We extend the models to account for the time-dependent droplet viscosity that results from non-isothermal conditions, and to account for non-zero final porosity. We also present a method to account for the initial distribution of radii of the pores interstitial to the liquid spheres, which allows the models to be used with no fitting parameters. We find a good agreement between the models and the data for times less than the capillary relaxation timescale. For longer times, we find an increasing discrepancy between the data and the model as the Darcy outgassing time-scale approaches the sintering timescale. We conclude that the decreasing permeability of the sintering system inhibits late-stage densification. Finally, we determine the residual, trapped gas volume fraction at equilibrium using X-ray computed tomography and compare this with theoretical values for the critical gas volume fraction in systems of overlapping spheres.

## 1. Introduction

The sintering of high-viscosity droplets to form a denser, connected mass is important in a range of industrial and natural scenarios, including the fabrication of ceramics [1], metals and glass, the welding of volcanic ash [2] and the vitrification of Iron Age fortification walls [3,4]. In each case, the dynamics may differ because the physical origins of the stresses that drive and oppose sintering may vary, and the materials are variably heterogeneous. We focus on what is commonly called ‘viscous sintering’—the sintering of two or more viscous droplets in the regime where interfacial tension drives fluid flow—which constitutes a viscous end-member of droplet coalescence problems. The viscous sintering problem has been studied extensively since the early theoretical works of Frenkel [5] and Mackenzie & Shuttleworth [6]. More recent studies have built on those works using both experimental [2,7–9] and theoretical constraints [10,11]. Implicit in models of surface tension-driven viscous sintering is that the liquid droplets are in the low Eötvös number and high Ohnesorge number regimes. The Eötvös number is given by

1.1 $$\text{Eo}=\frac{\rho gR^{2}}{\Gamma },$$

where *ρ* is the liquid density, *g* is gravitational acceleration, *R* is the radius of the liquid droplet and *Γ* is the surface tension. For Eo≪1, the surface tension stress dominates the gravitational stress acting on the droplet. The Ohnesorge number is given by

1.2 $$\text{Oh}=\frac{\mu }{\sqrt{\rho \Gamma R}},$$

where *μ* is the liquid viscosity. For Oh≫1, viscosity is sufficiently high that inertial effects resulting from the surface tension-driven motion can be neglected.

**Figure 1.**
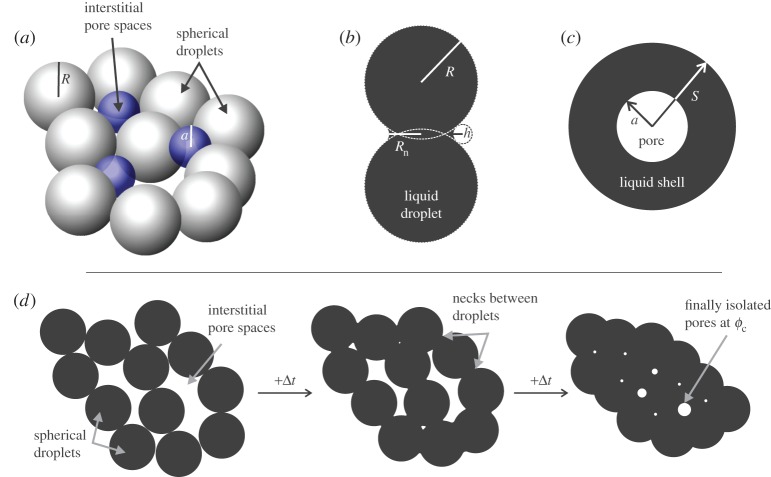
(*a*) A three-dimensional schematic representation of the initial system of viscous spherical droplets and effective interstitial, spherical pores. (*b*) The geometry used in the Frenkel [5] model (see §2b) here shown after some time when a neck of radius *R*_*n*_ has formed. (*c*) The geometry used in the Mackenzie & Shuttleworth [6] vented bubble model (see §2c and the electronic supplementary material). Here *S* is marked as the radius of the liquid shell surrounding the vented bubbles but is only used in the electronic supplementary material where the derivation of equation (2.13) can be found. (*d*) A two-dimensional schematic of how the system of spherical droplets is thought to evolve with time (here shown in time steps of +Δ*t*) toward equilibrium volume at *ϕ*=*ϕ*_*c*_). (Online version in colour.)

In theoretical studies of viscous sintering [2,6,7,9–12], the starting geometry is usually approximated as a packing of liquid spheres (figure 1). The progress of sintering is characterized by the evolution of the gas volume fraction *ϕ* as a function of time *t*, typically non-dimensionalized by a characteristic timescale [2,9,13], where the gas volume fraction is defined as the ratio of the volume of interstitial gas to the total volume of the sample or, equivalently, the ratio of the bulk sample density to the liquid density. Previous experimental work has been limited by the resolution of measurement of sample volumes such that uncertainties on *ϕ* preclude the validation of one model over another. An example of this resolution deficit is in the poor constraint of the final gas volume fraction: the critical porosity [14,15] at which the system is in volume equilibrium over timescales much longer than the experiments.

Silicate glass is well suited to the experimental constraint of viscous sintering because, when heated to temperatures in excess of the glass transition, the resultant undercooled liquid has a Newtonian viscosity that is sufficiently high [16–18] to permit the observation of the viscous processes over timescales that are amenable to laboratory investigation. It is also the material most pertinent to ceramic applications for which the viscous sintering process has been of particular interest [10]. High temperature optical dilatometry is suitable for the study of viscous sintering of glasses because it permits quantification of sample geometry *in situ* during heating [8,19] (electronic supplementary material, figure S1). Here we provide methods to convert two-dimensional observed sample geometries into high-resolution datasets for *ϕ*(*t*) and use these to test various theoretical models, described and developed in the next section.

## 2. Theoretical framework

### (a) Viscous sintering by interfacial tension

In liquid-phase sintering, droplets that share contacts undergo time-dependent coalescence driven by the interfacial tension between the liquid and the ambient fluid in the interstitial pore space. In the high Ohnesorge number regime (equation (1.2)), this process is dominated by the viscosity of the liquid droplets, and in the low Eötvös number regime (equation (1.1)), the stress driving flow arises from the excess surface pressure *P*, which is proportional to the local radii of curvature. For spherical liquid droplets, the two principle radii of curvature are equal to one another and to the radius of the droplet (figure 1) so this excess pressure is given by the Laplace general spherical solution *P*=2*Γ*/*R*.

The characteristic timescale associated with viscous flow, driven by interfacial tension, and neglecting inertia and buoyancy, is the capillary relaxation time λ

2.1 $$\lambda =\frac{\mu l}{\Gamma },$$

where *l* is a characteristic lengthscale. Normalizing the time of observation *t* by this timescale provides a non-dimensional capillary time $\bar{t}$ useful for characterizing the kinetics of viscous sintering.

Frenkel [5] proposes a model for the growth of necks between particles that share an initial contact, in which the initial radius of the droplet *R*_*i*_ is the characteristic lengthscale, yielding a dimensionless neck-formation time (denoted by subscript n)

2.2 $${\bar{t}}_{n}=\frac{t}{\lambda_{n}}=\frac{\Gamma }{\mu R_{i}}t.$$

Mackenzie & Shuttleworth [6] derive a model for the shrinking of pores interstitial to liquid droplets, in which the initial radius of the pore or bubble *a*_*i*_ is the characteristic lengthscale, yielding a dimensionless bubble relaxation time (denoted by subscript b)

2.3 $${\bar{t}}_{b}=\frac{t}{\lambda_{b}}=\frac{\Gamma }{\mu a_{i}}t.$$

In our experiments, and in many scenarios of practical interest, temperature is not constant, but is a function of time (electronic supplementary material, figure S2). Viscosity is a function of temperature, hence from equation (2.1) we see that the capillary relaxation timescale is also a function of temperature, hence also of time. The temperature dependence of surface tension is generally negligible for silicate liquids [20]. Expressing equation (2.2) in differential form, we can write an expression for the instantaneous variation in the dimensionless neck-formation time as a function of time

2.4 $$\frac{\text{d}{\bar{t}}_{n}}{\text{d}t}=\frac{1}{\lambda_{n}(t)}=\frac{\Gamma }{\mu (T)R_{i}}.$$

Integrating, we obtain an expression for the dimensionless neck-formation time for non-isothermal conditions

2.5 $${\bar{t}}_{n}=\int_{0}^{t}\frac{1}{\lambda_{n}(t)}\;\text{d}t=\frac{\Gamma }{R_{i}}\int_{0}^{t}\frac{1}{\mu (T)}\;\text{d}t.$$

Similarly, for dimensionless bubble relaxation time

2.6 $${\bar{t}}_{b}=\int_{0}^{t}\frac{1}{\lambda_{b}(t)}\;\text{d}t=\frac{\Gamma }{a_{i}}\int_{0}^{t}\frac{1}{\mu (T)}\;\text{d}t.$$

These integrals can be evaluated if *μ*(*T*) and *T*(*t*) are known. For isothermal conditions, equations (2.5) and (2.6) reduce to equations (2.2) and (2.3), respectively. These equations allow us to develop dimensionless forms of the Frenkel and the Mackenzie and Shuttleworth models in §2b,c, respectively, that are suitable for both isothermal and non-isothermal conditions.

### (b) The neck-formation model

Frenkel [5] derives a solution for the growth of the radius of a neck *R*_*n*_ forming between two liquid droplets of equal initial radius, as a function of time

2.7 $$R_{n}^{2}=\frac{3R_{i}\Gamma }{2\mu }t.$$

Kang [21] proposes that the external radius of curvature of the neck region *h* can be related to *R*_*n*_ and *R*_*i*_ by *h*≈*R*^2^_*n*_/(4*R*_*i*_) (figure 1*b*). Combining this approximation with equation (2.7), Kang [21] derives a linear shrinkage equation for spheres in series, cast as the length of the system *L* relative to the initial length *L*_*i*_

2.8 $$\frac{\Delta L}{L_{i}}=1-\frac{L}{L_{i}}\approx \frac{h}{R_{i}}=\frac{3\Gamma }{8\mu R_{i}}t.$$

Prado *et al.* [22] and Soares *et al.* [8] extend this analysis to the volumetric isotropic strain in an array of cubically packed monodisperse spheres, deriving a model for the gas volume fraction *ϕ* as a function of time

2.9 $$\phi =1+(\phi_{i}-1){\left(1-\frac{3\Gamma }{8\mu R_{i}}t\right)}^{-3},$$

where *ϕ*_*i*_ is the gas volume fraction at time *t*=0. Introducing the normalization $\bar{\phi }=\phi /\phi_{i}$, and using equation (2.2), we obtain a dimensionless form of equation (2.9)

2.10 $$\bar{\phi }=\frac{1}{\phi_{i}}+\left(1-\frac{1}{\phi_{i}}\right){\left(1-\frac{3}{8}{\bar{t}}_{n}\right)}^{-3}.$$

If the non-isothermal expression equation (2.5) is used to compute ${\bar{t}}_{n}$, then equation (2.10) can be applied to sintering under arbitrary thermal history.

### (c) The vented bubble model

Mackenzie & Shuttleworth [6] present an idealized model of sintering in which the interstitial, gas-filled pore space surrounding the droplets is represented as an array of spherical bubbles, evenly distributed throughout the liquid. Each bubble of radius *a* sits in a spherical liquid shell of radius *S*, and shrinks under the action of the surface tension between bubble and liquid (figure 1*c*). They derive an expression for the evolution of the bulk density of the bubble–shell unit as a function of time. In this scenario, the gas is assumed to be able to escape freely (despite the lack of physical escape routes) so we term this the ‘vented bubble’ model. Conceptually, the formulation is very similar to that used in studies of the growth of bubbles in magma [23] and, in the electronic supplementary material, we provide an alternative derivation of the vented bubble model, based on a bubble-growth model.

The Mackenzie & Shuttleworth [6] solution can be cast as a rate of change of gas volume fraction to give

2.11 $$\frac{\text{d}\phi }{\text{d}t}=-\frac{3\Gamma }{2\mu }{\left(N_{b}\frac{4\pi }{3}\right)}^{1/3}\phi^{2/3}{\left(1-\phi \right)}^{1/3},$$

where *N*_*b*_ is the bubble number density in the system. In this model *ϕ*→0 at t→∞. We find it convenient to recast *N*_*b*_ in terms of the initial gas volume fraction, which is more easily measured in practice than *N*_*b*_, via the equivalence $N_{b}4\pi a_{i}^{3}/3=\phi_{i}/(1-\phi_{i})$, to give

2.12 $$\frac{\text{d}\phi }{\text{d}t}=-\frac{3\Gamma }{2\mu a_{i}}{\left(\frac{\phi_{i}}{1-\phi_{i}}\right)}^{1/3}\phi^{2/3}{\left(1-\phi \right)}^{1/3},$$

which carries the implicit assumption that *N*_*b*_ is constant throughout the sintering process. As with the Frenkel [5] model, we can normalize *ϕ* by *ϕ*_*i*_ and use equation (2.3) to obtain a dimensionless form of equation (2.12)

2.13 $$\frac{d\bar{\phi }}{d{\bar{t}}_{b}}=-\frac{3}{2}{\left(\frac{1-\phi_{i}\bar{\phi }}{1-\phi_{i}}\right)}^{1/3}{\bar{\phi }}^{2/3}.$$

As before, if the non-isothermal expression (equation (2.6)) is used to compute ${\bar{t}}_{b}$, then equation (2.13) can be applied to sintering under arbitrary thermal history.

The differential equations above cannot be recast to give gas volume fraction as a simple function of time or temperature. However, if we make the simplifying assumption that *ϕ*≪1, so that equation (2.13) becomes

2.14 $$\frac{d\bar{\phi }}{d{\bar{t}}_{b}}=-\frac{3}{2}\frac{{\bar{\phi }}^{2/3}}{{\left(1-\phi_{i}\right)}^{1/3}},$$

then this simplified form can be integrated subject to the initial conditions $\bar{\phi }$ at ${\bar{t}}_{b}=0$ to give

2.15 $$\bar{\phi }={\left(1-\frac{1}{2{\left(1-\phi_{i}\right)}^{1/3}}{\bar{t}}_{b}\right)}^{3},$$

which represents the non-isothermal, dimensionless extension of the Mackenzie & Shuttleworth [6] derivation when the initial gas volume fraction is assumed to be small.

### (d) The exponential approximation

Chiang *et al.* [24] make the observation that the relationship *N*_*b*_4*πa*^3^/3=*ϕ*/(1−*ϕ*) allows the Mackenzie & Shuttleworth [6] model (equation (2.11)) to be simplified to give

2.16 $$\frac{\text{d}\phi }{\text{d}t}=-\frac{3\Gamma }{2\mu a}\phi .$$

Note that this formulation uses the time-dependent bubble radius and gas volume fraction (*a* and *ϕ*) rather than the initial radius and gas volume fraction (*a*_*i*_ and *ϕ*_*i*_) that yield equation (2.12). Consequently, non-dimensionalization requires the use of a modified bubble relaxation time, in which the bubble radius is a function of time. Following the approach outlined in §2a, we couch ${\bar{t}}_{b}(t)$ in differential form, then integrate to obtain

2.17 $${\bar{t}}_{b}^{*}=\int_{0}^{t}\frac{1}{\lambda_{b}(t)}\;\text{d}t=\Gamma \int_{0}^{t}\frac{1}{\mu a(t)}\;\text{d}t,$$

where the superscript asterisk indicates that *a* is a function of time. Note that this form also accounts for non-isothermal conditions if *μ* is also treated as a function of time. The dimensionless form of equation (2.16) is then

2.18 $$\frac{d\bar{\phi }}{d{\bar{t}}_{b}^{*}}=-\frac{3}{2}\bar{\phi },$$

permitting an analytical solution with an exponential dependence of $\bar{\phi }$ on ${\bar{t}}_{b}^{*}$,

2.19 $$\bar{\phi }=\exp\left(-\frac{3}{2}{\bar{t}}_{b}^{*}\right).$$

This relationship is of little practical use because *a*(*t*) is not known *a priori*. Nonetheless, equation (2.19) and variations on a non-isothermal formulation have been widely used to describe sintering of liquid droplets [2,7,8,19,25], but without acknowledgement of the implicit approximation that *a*=*a*_*i*_, for all *t*. We explicitly adopt this approximation and will assess its value against our experimental data later, in §4.

### (e) Extension to account for non-zero final gas volume fraction

It is a common observation that the gas volume fraction of a sintered mass does not reach zero, but approaches a final gas volume fraction *ϕ*_*f*_ [2,7,9,25–27]. This is discussed further in §2f, but we find it convenient here to accommodate this observation by substituting *ϕ*−*ϕ*_*f*_ in place of *ϕ* and *ϕ*_*i*_−*ϕ*_*f*_ in place of *ϕ*_*i*_ in our system of equations. The normalization of *ϕ* then becomes ${\bar{\phi }}^{*}=(\phi -\phi_{f})/(\phi_{i}-\phi_{f}$), and equations (2.13), (2.15) and (2.19) become, respectively,

2.20 $$\begin{array}{rl} \frac{\text{d}{\bar{\phi }}^{*}}{\text{d}{\bar{t}}_{b}} & =-\frac{3}{2}{\left(\frac{1-(\phi_{i}-\phi_{f}){\bar{\phi }}^{*}}{1-(\phi_{i}-\phi_{f})}\right)}^{1/3}{\bar{\phi }}^{{*}^{2/3}}, \end{array}$$

2.21 $$\begin{array}{rl} {\bar{\phi }}^{*} & ={\left(1-\frac{1}{2{\left(1-(\phi_{i}-\phi_{f})\right)}^{1/3}}{\bar{t}}_{b}\right)}^{3} \end{array}$$

2.22 $$\begin{array}{rl} \text{and}\;{\bar{\phi }}^{*} & =\text{exp}\left(-\frac{3}{2}{\bar{t}}_{b}^{*}\right). \end{array}$$

The empirical adjustment that we make is not strictly consistent with the derivation of the models; nonetheless, we expect that any violation of the model assumptions will be inconsequential for small *ϕ*_f_, and propose that any consequent loss of fidelity will be outweighed by the advantage gained from capturing the non-zero final porosity. We leave the models to be tested against data later, in §4.

### (f) The initial bubble radius a_*i*_

The radii of initially spherical glass spheres are trivial to constrain using a variety of techniques (see §3) providing constraint of the lengthscale *R*_*i*_ for use with the neck-formation model [5]. However, the lengthscale *a*_*i*_ that appears in the vented bubble model [6] and our extensions thereof in §2c,d is a less easy-to-constrain parameter. However, Torquato [28] and Torquato & Avellaneda [29] provide a rigorous expression for a mean pore size *a* occurring between particles in arbitrary packing. Their scheme can be cast for a packing of completely impenetrable ‘hard’ spheres: an arrangement identical to our initial case of packed glass beads (figure 1*a*). This is given in the form of a cumulative probability density *F*(*ζ*) of the pore size distribution for which *ζ*=*a*/*R*

2.23 $$F(\zeta )=\frac{E_{V}(\zeta )}{\phi },$$

where *E*_*V*_(*ζ*) is a pore nearest-neighbour exclusion probability function. In our system, this is a conceptual tool akin to finding the expected fraction of space available to a pore of radius *a*. To solve for *E*_*V*_ is a non-trivial problem that has received significant attention [28,29]. A validated expression for *E*_*V*_ as a function of *R* is given by Torquato [28] based on Torquato & Avellaneda [29] and reproduced here for completeness, where we cast it in terms of the gas volume fraction *ϕ*

2.24 $$E_{V}(\zeta )=\phi \text{exp((}\phi -1\text{)[}y_{0}{\left(1+\zeta \right)}^{3}+3y_{1}{\left(1+\zeta \right)}^{2}+12y_{2}(1+\zeta )+y_{3}]).$$

Equation (2.24) is valid for *ζ*≥0 and contains coefficients *y*_*n*_ which are given by

2.25 $$\begin{array}{rl} y_{0} & =\frac{2-\phi +{\left(1-\phi \right)}^{2}-{\left(1-\phi \right)}^{3}}{\phi^{3}},\\ y_{1} & =\frac{\left(1-\phi )(3{\left(1-\phi \right)}^{2}+4\phi -7\right)}{2\phi^{3}},\\ y_{2} & =\frac{{\left(1-\phi \right)}^{2}(1+\phi )}{2\phi^{3}}\\ \text{and}\;y_{3} & =-(y_{0}+3y_{1}+12y_{2}). \end{array}\}$$

The *n*th moment of the probability density function of *ζ*, termed 〈*ζ*^*n*^〉, is then related to the cumulative probability density function *F*(*ζ*) in equation (2.23) by integrating as follows:

2.26 $$\langle \zeta^{n}\rangle =n\int_{0}^{\infty }\zeta^{n-1}F(\zeta )\;\text{d}\zeta ,$$

hence the mean (i.e. *n*=1) value of 〈*a*〉 is *a*=〈*ζ*〉/〈*R*〉.

Equation (2.23)–(2.26) can be used to find *a* in the monodisperse limit of *R*. Torquato [28] describes the solution of Lu & Torquato [30], which further constrains a polydisperse solution which is again validated against data and reproduced here for completeness. In this form, the pore nearest-neighbour exclusion probability function is the polydisperse *e*_*V*_(*ζ*) and is

2.27 $$e_{V}(\zeta )=\phi \;\text{exp}\left(2S(\phi -1)\left[\frac{z_{0}}{8}{\left(1+\zeta \right)}^{3}+\frac{z_{1}}{4}{\left(1+\zeta \right)}^{2}+\frac{z_{2}}{2}(1+\zeta )\right]\right),$$

for which *S* is the ratio of the specific surface of the polydisperse system to that of the monodisperse system at the same value of *ϕ*. *S* is given by

2.28 $$S=\frac{\left\langle R^{2}\right\rangle }{\left\langle R^{3}\right\rangle }\langle R\rangle ,$$

where again 〈*R*^*n*^〉 is the *n*th moment of the probability density function of the particle radius distribution. As before, the coefficients *z*_*n*_ are given by specific solutions, here with a dependence on *S* and 〈*R*^*n*^〉

2.29 $$\begin{array}{rl} z_{0} & =\frac{4\phi {\left\langle R\right\rangle }^{2}[\phi +3S(1-\phi )]+8\langle R^{2}\rangle {\left[S(1-\phi )\right]}^{2}}{\phi^{3}\langle R^{2}\rangle },\\ z_{1} & =\frac{6\phi {\left\langle R\right\rangle }^{2}+9S\langle R^{2}\rangle (1-\phi )}{\phi^{2}\langle R^{2}\rangle }\\ \text{and}\;z_{2} & =\frac{3}{\phi }. \end{array}\}$$

To arrive at the polydisperse solution for *a* as a function of *R*, the same method as the monodisperse limit is applied but where *E*_*V*_(*ζ*) in equation (2.23) is replaced by *e*_*V*_(*ζ*) and equation (2.26) remains unchanged.

An example of a polydisperse limit is the Schulz distribution [31] for a polydispersivity factor *m*=0. This is found by relating 〈*R*^*n*^〉 to *m* by 〈*R*^*n*^〉=〈*R*〉^*n*^(*m*+*n*)!/[*m*!(*m*+1)^*n*^] such that when *m*=0 the particle size distribution is heavily weighted to small particle sizes and with a broad tail at the high particle size limit. See §4 for application of these constraints to our system.

### (g) The concept of volume equilibrium

In the above sections, we have explored and developed non-dimensional solutions to the main sintering models for the low Eötvös, high Ohnesorge number regime in §2b–d, and presented constraint of the pore size as a function of the initial droplet size distribution in §2f. Finally, we find it useful to constrain the final gas volume fraction at which the system is in volume equilibrium. That is, the percolation threshold *ϕ*_*c*_ at which the gas volume fraction becomes disconnected and forms isolated bubbles suspended in the liquid. Sintering must halt at *ϕ*=*ϕ*_*c*_ because at this point the bubbles are no longer ‘vented’ such that the gas permeability *k*→0 and pressure equilibrium between the now-isolated bubble and the liquid is established. A period of rounding of the bubble over the capillary timescale, equation (2.1), will occur but the gas volume fraction *ϕ* should remain in equilibrium (although the bubbles may rise out of the system buoyantly over longer timescales). This *ϕ*_*c*_ arises from percolation theory and is the same parameter we account for empirically via §2e [14,15,28]. Both Elam *et al.* [14] and Kertész [15] constrained values of *ϕ*_*c*_ for systems of randomly located monodisperse overlapping spheres that are in mutual agreement regardless of the sphere size used. This is close to, albeit statistically different from, the polydisperse *ϕ*_*c*_ found for the two distributions of droplets investigated by Rintoul [32]. The values for *ϕ*_*c*_ found by Elam *et al.* [14], Kertész [15] and Rintoul [32] are collated in table 2 for comparison with our experimental data. All these studies use numerical models that randomly place spheres that are permitted to fully overlap. In our system, this can be viewed as the initially spherical liquid droplets encroaching into one another with time until *ϕ*=*ϕ*_*c*_. We will compare the predicted values of *ϕ*_*c*_ with our data in §5b.

## 3. Experimental materials and methods

### (a) Material properties and experimental method

**Figure 2.**
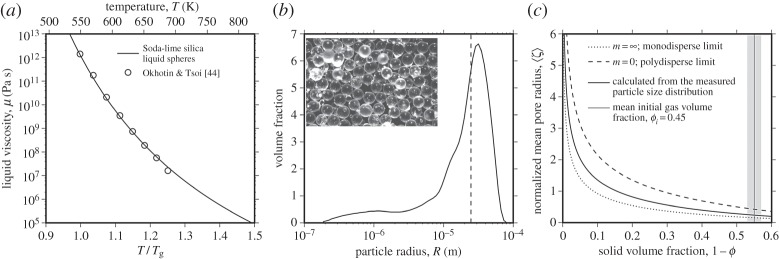
Properties and geometry of the experimental material used in this study. (*a*) The temperature dependence of the Newtonian liquid viscosity for the soda-lime silica liquid droplets normalized to the glass transition onset *T*_g_ determined at 10 K min^−1^ linear heating rate to be approximately 824 K [2]. The curve is a fit to the data using the non-Arrhenian Vogel–Fulcher–Tammann expression. (*b*) The particle size distribution for the liquid droplets with the mean radius 〈*R*〉 marked as a vertical dashed line. *Inset* is an optical micrograph of the glass spheres used to produce the liquid droplets here sieved to the largest size fraction used for image quality. The base of the image is 800 *μ*m. (*c*) The normalized mean pore radius 〈*ζ*〉 calculated from equations (2.23) and (2.26)–(2.29) as a function of the solid volume fraction used to estimate 〈*a*_*i*_〉 when *ϕ*=*ϕ*_*i*_ (see §2f).

To assess the viscous sintering of droplets we use populations of glass spheres which, when heated above their glass transition temperature, are metastable undercooled liquids. As an experimental starting material, we use soda-lime silica glass spheres (Spheriglass^®^ A-glass microspheres product number 2530, Potters Industries Inc.). This material has been shown to have a reproducible glass transition onset *T*_g_ at a heating rate of 10 K min^−1^ of approximately 824 K and a stable mass over the temperature range 270–1670 K [2]. The temperature dependence of the Newtonian liquid viscosity above *T*_g_ can be fitted using a Vogel–Fulcher–Tammann equation of the form *μ*=*A*+*B*/(*T*−*C*) for which the fitted *A*, *B* and *C* parameters are −2.63, 4303.36 and 530.60, respectively [2], and *T* is in units of Kelvin (figure 2*a*). We note that when shear stresses acting on silicate liquids are large, non-Newtonian effects have been measured [33]; however, the shear stresses imposed by surface tension are sufficiently small that a Newtonian viscosity is sufficient to describe the rheology. Surface tension is negligibly dependent on temperature; we use the value *Γ*=0.3 N m^−1^ for dry silicate liquids [34,35].

The particle radii were measured, after sieving to size fractions below approximately 63 *μ*m, using a Beckman Coulter LS^TM^ 230 laser refraction particle size analyser with a measuring range 0.375–2000 *μ*m. The mean radius 〈*R*〉 was then calculated to be 24.7±1.6 *μ*m (figure 2*b*). Using the particle size distribution, we can calculate a predicted mean of the bubble radii interstitial to the polydisperse particles using equations (2.23) and (2.26)–(2.29), yielding 〈*a*_*i*_〉 of 5.9±0.53 *μ*m (figure 2*c*).

The starting samples for our experiments were free-standing cylinders of beads. The cylinders, approximately 3 mm tall with radius approximately 1.465 mm, were formed by filling a die with glass beads, compacting with a pressure-gauged push-rod, and extracting the sample onto a ceramic plate. These samples were loaded into a Hesse Instruments EM-201 optical dilatometer, which consists of a halogen lamp, tube furnace and camera in series such that the camera is able to record a cross-sectional image of the sample (electronic supplementary material, figures S1 and S2). Samples were heated to different experimental temperatures *T*_0_ in the range 841 to 1164 K. Software developed by Hesse Instruments processes silhouettes of the sample, which are then converted to binary images collected at 1 Hz sampling rate. The cross-sectional area, height and width of the sample silhouette is computed in real time and continuously recorded in units of pixels. Samples were held at *T*_0_ until volume equilibrium was attained.

A type-S thermocouple is embedded in the ceramic sample holder within 1.5 mm of the base of the sample and is used to monitor the experimental temperature, calibrated to within 1.6 K. Low heating rates of 10 K min^−1^ were used to ensure nominal thermal equilibrium of the sample [2] during heating. Following heating, samples were held within ±2 K of *T*_0_.

### (b) Isotropic shrinkage of a cylinder

Our samples are initially cylindrical and shrink with time. If we first assume that the geometry remains cylindrical during shrinkage, we can determine the volume as a function of time as follows. A cylinder of height *L* and radius *r* that has shrunk isotropically by a factor *α*=*L*/*L*_*i*_=*r*/*r*_*i*_ has volume *V* =*α*^3^*πr*^2^_*i*_*L*_*i*_ and maximum cross-sectional area in the *r*–*L* plane *A*=2*α*^2^*r*_*i*_*L*_*i*_, where the subscript *i* indicates the initial dimensions. It follows that *V* =*V*_*i*_(*A*/*A*_*i*_)^3/2^, hence continuous measurement of *A* permits the cylindrical volume to be computed for all times. Volume can be converted to gas volume fraction by *ϕ*=1−*m*_*i*_/(*ρV*), where *ρ* is the liquid density and *m*_*i*_ is the mass of the system assuming that the only contribution to *m*_*i*_ is from the liquid and that no mass changes occur with time. Errors associated with this measurement arise from pixel resolution and the method of pixel size calibration.

### (c) Axisymmetric volumes by the solid of rotation

The volume of the sample can also be calculated from its silhouette by treating it as a solid of rotation (electronic supplementary material, figure S2*a*), and integrating the radial distance from the axis of symmetry to the sample edge as a function of vertical position, to give $V=\int_{0}^{L}\pi r^{2}\;\text{d}y$. This approach will give greater accuracy than the cylinder method if there are vertical variations in the radial shrinkage factor. The edges are detected from binary images using a Canny edge detector algorithm (electronic supplementary material, figure S2*b*) and, rastering up the image in 1-pixel horizontal slices, the width of the cylinder is measured for each slice; the radius of the cylinder *r* is taken as half the width of the slice in pixels. The volume of the cylinder calculated is in voxels, which we calibrate against the final volume of the sample (see §4a). Knowing that the glass density is *ρ* and sample mass is *m*_*i*_, we can convert final volume to final gas volume fraction *ϕ*_*f*_. Once *ϕ*_*f*_ is determined, we can obtain the time-dependent gas volume fraction using *ϕ*=1−(*V*_*f*_/*V*)(1−*ϕ*_*f*_), where *V*_*f*_ and *V* can remain in voxel units.

## 4. Results and interpretation

### (a) Calibrating time-dependent porosity curves

**Figure 3.**
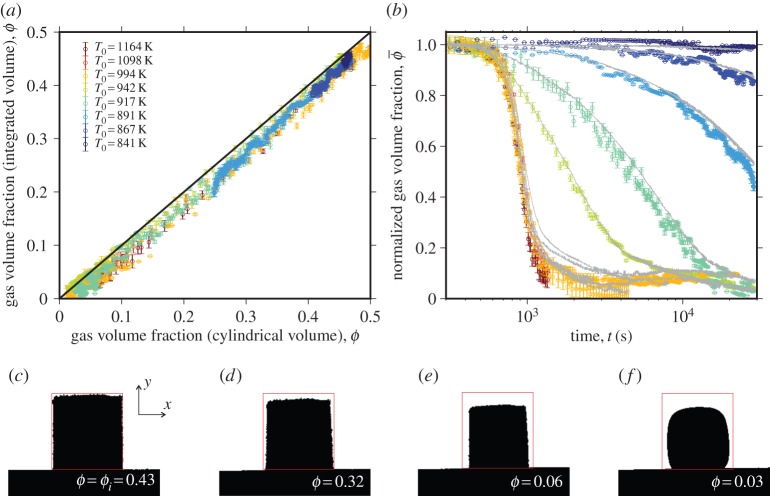
The evolution of the gas volume fraction of the cylinders of packed liquid droplets. (*a*) Comparison of the techniques of determining the gas volume fraction for all experimental data demonstrating that cylindrical approximations are valid. (*b*) The gas volume fraction normalized to the initial value as a function of dimensional time after the calorimetric glass transition onset. The data points are the results of volume integration technique described in §3c applied to the images captured using optical dilatometry and the continuous curves are the results of using the cylindrical approximation in §3b. (*c*–*f*)Example binary images from the optical dilatometry camera for which a rectangular approximation of the initial sample size is marked by a box. (Online version in colour.)

We compare the time-dependent porosity data determined via the methods described in §3b,c (figure 3*a*). The cylindrical assumption (§3b) consistently overestimates the porosity by an average of 4.7% compared with the solid of rotation method (§3c). Throughout the analysis in this study, we use the data derived from the more general method §3c but point out here that the cylindrical assumption of §3b, which is commonly used in other studies [2,8,19], systematically overestimates the majority of the sintering process, albeit with a small error.

To calibrate the volumes computed we used X-ray computed microtomography to measure the final gas volume fraction in selected post-experimental samples that had attained volume equilibrium. Samples were mounted onto an alumina rod and clamped to the rotation rig. Images were captured using a Phoenix Nanotom E system operating at 80 kV using a 0.1 mm Cu filter to reduce beam-hardening. Images were reconstructed from 1440 projections using standard proprietary filtered back projection algorithms and the resolution (pixel sizes) range from 1.42 to 1.59 μm. Image visualization and analysis was performed using Avizo^TM^. Pore volumes were segmented from the central region of each sample to avoid edge effects. Segmentation was performed using a standard gradient-based algorithm using the moments of the intensity distribution. All pores with volumes less than 125 voxels were discarded from the analysis as below this value the error on absolute volumes exceeds 5% [36] and these objects only comprise approximately 0.06–0.18 vol.% of the sample. Pore volumes were calculated for remaining segmented features. We note that there is a negligible average difference of approximately 0.5 vol.% in the computed pore volumes if we take the three-dimensional data from the whole sample rather than from a cropped sub-volume.

We expect that the actual value of *ϕ*_*f*_ measured at room temperature will be slightly lower than that measured at *T*_0_ because the bubbles may shrink in response to thermal contraction of the gas on cooling from *T*_0_ to *T*_g_. We can estimate the magnitude of this contraction by using the ratio of the ideal equilibrium gas volumes at temperatures *T*_0_ and *T*_g_ which, assuming the number of moles of gas are constant in these isolated pores, reduces to *T*_0_/*T*_g_. For our experimental temperatures this equates to a maximum fractional error in *ϕ* on cooling of approximately 0.01. We do not correct the curves and rather consider this a minor source of error in our determination of the final porosity. We additionally assume that the contraction of the liquid and glass is negligible on heating or cooling [37] compared with the contraction of the gas, or equivalently, the density of the liquid and glass is taken as constant. This assumption is justified because the thermal expansion coefficient for silicate glass is approximately 10^−5^ K^−1^ [38]; hence the variation in glass density between room temperature and *T*_g_ on heating or cooling is less than 1%, which is small compared with density changes resulting from sintering.

### (b) Testing models using best-fit droplet and pore radii

After applying the solid of rotation to obtain time-dependent volumes of high temperature experimental samples *in situ* in voxels and subsequently converting these to *ϕ*(*t*) (§3), we obtain the data presented in figure 3*b*. The curves all show a rapid onset of *ϕ* decay followed by a long tail at high values of *t*. While the shape of the curves is similar across all *T*_0_, the absolute rate of this process is systematically dependent on *T*_0_.

We use our experimental results to test the theoretical models presented in §2. In all cases, the models are tested in dimensionless form, which necessitates transforming the raw datasets—i.e. *ϕ*(*t*) for each experimental run—into $\bar{\phi }({\bar{t}}_{n})$, $\bar{\phi }({\bar{t}}_{b})$ or ${\bar{\phi }}^{*}({\bar{t}}_{b})$ depending on the model to be tested. Gas volume fraction *ϕ* is trivially non-dimensionalized as $\bar{\phi }=\phi /\phi_{i}$ or ${\bar{\phi }}^{*}=(\phi -\phi_{f})/(\phi_{i}-\phi_{f})$. Where non-isothermal behaviour can be ignored, equation (2.2) or (2.3) is used to non-dimensionalize *t* as ${\bar{t}}_{n}$ or ${\bar{t}}_{b}$, respectively. In either case, viscosity is calculated from the relationship given in §3a, and surface tension *Γ* is constant at *Γ*=0.3 N m^−1^. If equation (2.2) is used, the initial droplet radius *R*_*i*_ is either taken from the measured particle size distribution, or treated as a fitting parameter. If equation (2.3) is used, the initial pore radius *a*_*i*_ is either calculated from *R*_*i*_ following the approach outlined in §2f, or treated as a fitting parameter.

Where non-isothermal behaviour is important, non-dimensionalizing *t* is slightly more complex. The temperature–time data for the run is used to calculate ${\bar{t}}_{n}$ or ${\bar{t}}_{b}$ via equation (2.5) or (2.6), as required. As before, μ is calculated from the relationship given in §3a, and surface tension *Γ* is constant. If equation (2.5) is used, the initial droplet radius *R*_*i*_ is either taken from the measured particle size distribution, or treated as a fitting parameter. If equation (2.6) is used, the initial pore radius *a*_*i*_ is either calculated from *R*_*i*_ following the approach outlined in §2f, or treated as a fitting parameter.

In §4b, we allow the initial droplet radius *R*_*i*_ and initial pore radius *a*_*i*_ to vary freely, as fitting parameters; in §4c, we constrain these radii based on measured particle size distributions. This two-step analysis allows us to assess the consistency of each model across the large range of liquid viscosities investigated before generalizing the models without any fitting procedure.

#### (i) The neck-formation model

**Figure 4.**
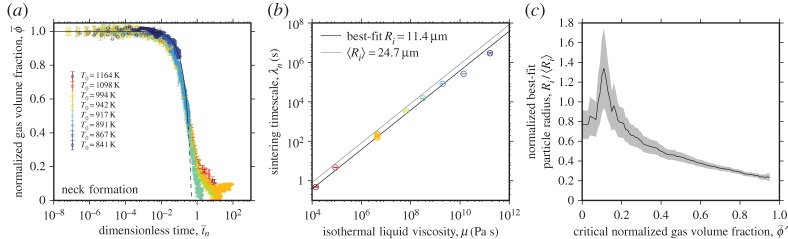
Testing the neck-formation model [5]. (*a*) Experimental data for $\bar{\phi }$ as a function of ${\bar{t}}_{n}$ for each experimental run; λ_n_ is a fitted parameter. The neck-formation model (equation (2.10)) is shown for comparison (solid line) and dashed below ${\bar{\phi }}^{\prime }$, here taken as 0.44. (*b*) The fitted timescale λ_n_ compared with the isothermal liquid viscosity μ at the experimental temperature *T*_0_ corresponds to an average best-fit *R*_i_ of 11.4 μm shown here for $\bar{\phi }$. The curve corresponding to the value of 〈*R*_i_〉 estimated using the particle size distribution (figure 3) is shown for comparison. (*c*) Dependence of the calculated initial droplet radius *R*_i_ (determined from λ_n_ via equation (2.5), and normalized by measured 〈*R*_i_〉) on the gas volume fraction at which the neck-formation stage of sintering ends ${\bar{\phi }}^{\prime }$ (see §4a(i) for explanation of approach). The grey-shaded area denotes the error on the determination of *R*_i_. (Online version in colour.)

For each dataset, the best fit of the neck-formation model (equation (2.10)) is found with *R*_*i*_ as a free fitting parameter using a least-squares regression procedure. When the experimental time *t* is normalized using the best-fit value of *R*_*i*_ obtained, the data collapse to close to a single curve. Compared with the model itself (equation (2.10)), this produces a moderate fit for all temperatures (figure 4*a*). Using equation (2.2) and the definitions of the isothermal viscosity μ and the surface tension *Γ* discussed, a least-squares regression method can be used to fit the linear relationship across all temperatures in figure 4*b*, which determines a best-fit characteristic radius *R*_*i*_. For all values of μ, this yields an estimated average *R*_*i*_=11.4±1.2 μm (black curve figure 4*b*) via equation (2.2). This *R*_*i*_ compares favourably with the mean radius from the measured particle size distribution of 〈*R*_*i*_〉=24.7 μm. When a single best-fit characteristic *R*_*i*_ is found for all isothermal temperatures, the coefficient of determination is 0.984 (figure 4*b*; table 1).

**Table 1.** Mean fitted values across all experimental temperatures.

model	*R*_i_ (μm)	coefficient of determination	*a*_*i*_;*ϕ*→0 (μm)	coefficient of determination	*a*_*i*_;*ϕ*→*ϕ*_*f*_ (μm)	coefficient of determination
neck formation^a^	11.45±1.25^d^	0.983	—	—	—	—
vented bubble^b^	—	—	11.01±1.18	0.993	9.55±0.93	0.994
small *φ*	—	—	12.68±1.29	0.994	10.85±1.01	0.995
approximation^c^						
exponential	—	—	9.29±0.86	0.995	7.92±0.64	0.996
approximation^c^						

^a^Frenkel [5].

^b^Mackenzie & Shuttleworth [6].

^c^Extended models; this study.

^d^Example for ${\bar{\phi }}^{\prime }=0.25$.

The model of Frenkel [5] tested here is based on the formation of necks between liquid droplets and as such is expected to describe the early part of the sintering process better than the later part. This is confirmed by figure 4*a*, in which the model curve decays to $\bar{\phi }=0$ prior to the observed tail of the process. Therefore, if this model has validity, it is likely only for the initial part of the sintering process, when mass transport of liquid is dominantly in necks between particles.

In order to explore this further, we repeat the fitting process multiple times for all datasets, each time fitting a slightly greater fraction of the data. In each case, we start the fit at $\bar{\phi }=1$ (i.e at *t*=0) and fit to a volume fraction ${\bar{\phi }}^{\prime }$, deriving a best-fit value for *R*_i_ for that fraction of the data. Figure 4*c* plots *R*_i_ against ${\bar{\phi }}^{\prime }$, with *R*_i_ normalized by 〈*R*_i_〉, such that a value of *R*_i_/〈*R*_i_〉 closer to 1 indicates a good fit between computed and measured particle radius. The plot demonstrates that the model and data are in closest agreement when we fit only the early sintering data, and that the fit worsens as more data are included in the fit. The data presented in figure 4*a*,*b* were calculated using ${\bar{\phi }}^{\prime }=0.44$, which, for our *ϕ*_*i*_, corresponds to *ϕ*=0.2, which is the value above which Prado *et al.* [7] claim the Frenkel model is applicable. However, here ${\bar{\phi }}^{\prime }=0.44$ is intended to be illustrative rather than diagnostic of the efficacy of this model.

#### (ii) The vented bubble model and exponential approximation

**Figure 5.**
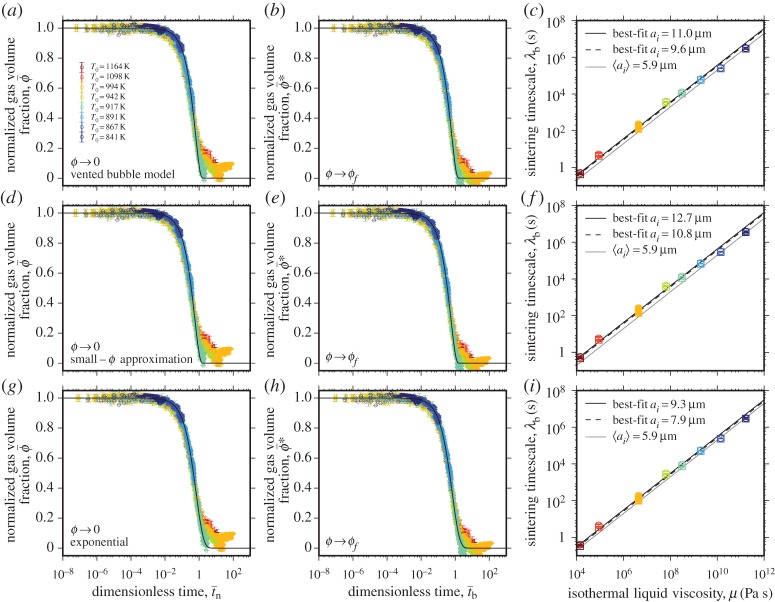
Testing the models in which the characteristic lengthscale is related to the pores and not the droplets and is a fitting parameter. (*a*–*c*) Testing the vented bubble model [6] when (*a*) $\bar{\phi }=0$ at ${\bar{t}}_{b}\to \infty$ using equation (2.13) and (*b*) when $\bar{\phi }=\phi_{f}$ at ${\bar{t}}_{b}\to \infty$ using equation (2.20). (*d*–*f*) Testing the small *ϕ* approximation of the vented bubble model (*d*) when $\bar{\phi }=0$ at ${\bar{t}}_{b}\to \infty$ using equation (2.15) and (*e*) when $\bar{\phi }=\phi_{f}$ at ${\bar{t}}_{b}\to \infty$ using equation 2.21. (*g*–*i*) Testing the approximate exponential model (*g*) when $\bar{\phi }\to 0$ using equation (2.19) and (*h*) when $\bar{\phi }\to \phi_{f}$ using equation (2.22). (*c*), (*f*) and (*i*) show the fitted timescale λ_*b*_ compared with the equivalent isothermal liquid viscosity μ for each model, respectively, and for comparison, the curve corresponding to the value of 〈*a*_*i*_〉 estimated from the particle size distribution using equations (2.23) and (2.26)–(2.29) is shown. (Online version in colour.)

**Figure 6.**
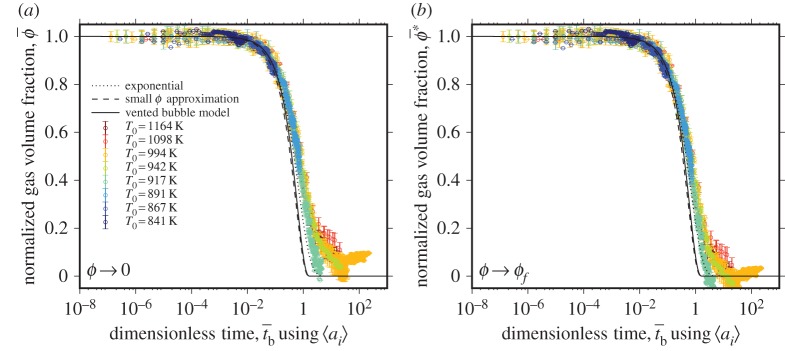
(*a*,*b*) The dimensionless results for $\bar{\phi }$ with ${\bar{t}}_{b}$ when the lengthscale used to normalize the experimental time *t* is the predicted *a*_*i*_ from equations (2.23) and (2.26)–(2.29) using no fitting procedure. All data collapse to a unique description with excellent agreement with the vented bubble model modified from Mackenzie & Shuttleworth [6] and the small *ϕ* approximation thereof. The high temperature data deviate at high values of ${\bar{t}}_{b}$ (see §4). (*a*) $\bar{\phi }=0$ at ${\bar{t}}_{b}\to \infty$; (*b*) $\bar{\phi }=\phi_{f}$ at ${\bar{t}}_{b}\to \infty$. (Online version in colour.)

Next, we test the vented bubble model, which was modified after Mackenzie & Shuttleworth [6] in §2c. This model uses the dimensionless time ${\bar{t}}_{b}$ in which time *t* is normalized to the bubble capillary timescale λ_*b*_. The model, which is solved numerically, can either go to $\bar{\phi }=0$ (equation (2.13); figure 5*a*) or to $\bar{\phi }=\phi_{f}$ (equation (2.20); figure 5*b*). In the former case, the best-fit timescale λ_*b*_ for all experiments yields a best-fit bubble radius *a*_*i*_ of 11.0±1.2 μm and in the latter case, a best-fit *a*_*i*_ of 9.6±0.9 μm, both of which compare favourably with the 〈*a*_*i*_〉 value of 5.9 μm computed following the approach described in §2f (equations (2.23) and (2.26)–(2.29)). To fit for the characteristic *a*_*i*_, we use a least-squares approach and equation (2.3). Finding a common *a*_*i*_ for all experimental temperatures yields a coefficient of determination of 0.993 (figure 5*c*; table 1). The agreement between the fit *a*_*i*_ and the measured 〈*a*_*i*_〉 is manifest in the success of the collapse to a single curve of $\bar{\phi }$ with ${\bar{t}}_{b}$ (figures 5*a* and 6*b*). The final gas volume fraction *ϕ*_*f*_ refers to the minimum observed value that is confirmed by X-ray micro computed tomography (§4a) and is measured to be approximately 0.03 (table 2).

**Table 2.** Equilibrium gas volume fraction, *ϕ*_*c*_.

	1−*ϕ*_c_	
references	monodisperse	polydisperse
Elam *et al.*[14]	0.968±0.004	—
Kertesz [15]	0.966±0.007	—
Rintoul [32]	0.9699±0.0003	0.9713±0.0005
this work	—	0.97±0.008

The Mackenzie & Shuttleworth [6] model yields an analytical approximation when *ϕ*_i_≪1 by equation (2.15) and (2.21). We test this against the experimental data in figure 5*d*,*e*. Whether $\bar{\phi }$ goes to zero (figure 5*d*) or to the empirically observed *ϕ*_*f*_ (figure 5*e*), the best-fit *a*_i_ is within error of the estimated 〈*a*_i_〉 for all experimental values of μ (figure 5*f*). For our samples, for which the average *ϕ*_i_=0.45±0.02, the small *ϕ* approximation provides an excellent collapse of the data to a single curve for a common characteristic radius *a*_i_, with a coefficient of determination of 0.994 (table 1).

Finally, we test the commonly used [2,7,8,19,24] exponential approximation of the vented bubble model (equations (2.19) and (2.22)) as described in §2d, where we note the implicit assumption that bubble radius is independent of time. Despite this assumption, which must, in reality, be violated, the results of fitting for the timescale λ_*b*_ for both the $\bar{\phi }\to 0$ and $\bar{\phi }\to \phi_{f}$ conditions are very close to each other (figure 5*g*,*h*) and almost indistinguishable from those of the small *ϕ* approximation, resulting in average best-fit radii in excellent agreement with 〈*a*_i_〉 and a coefficient of determination of 0.995 (table 1).

All best-fit radii and coefficients of determination are summarized in table 1.

### (c) Testing models without fitting

In figure 6, we show all data from figure 3 normalized by the capillary timescale λ_*b*_ in which the lengthscale is the radius of the bubbles interstitial to the mean of the particles 〈*a*_i_〉 estimated using Equations (2.23) and (2.26)–(2.29), which we have shown provides a reasonable approximation to the lengthscale controlling the best-fit timescales across all experiments (figure 5). This permits all data to be collapsed to a single description of $\bar{\phi }$ as a function of dimensionless time ${\bar{t}}_{b}$ without any fitting parameters. Furthermore, it permits us to directly compare the three models that are based on interfacial tension around bubbles interstitial to the particles: (i) the vented bubble model modified from Mackenzie & Shuttleworth [6]; (ii) the small *ϕ* approximation of the vented bubble model; and (iii) the exponential approximation [24]. We show both the solutions when $\bar{\phi }=0$ at ${\bar{t}}_{b}\to \infty$ (figure 6*a*) and when $\bar{\phi }=\phi_{f}$ at ${\bar{t}}_{b}\to \infty$ (figure 6*b*). The vented bubble model modified from Mackenzie & Shuttleworth [6] and the small *ϕ* approximation thereof are almost indistinguishable from one another for the values of *ϕ*_i_ represented by our samples, and both provide a good agreement with the data. However, we note that there is a systematic deviation from the predicted behaviour at values of ${\bar{t}}_{b}$ approaching unity. This phenomenon is discussed in §5. Nevertheless, the vented bubble model well captures the data across a huge range of experimental temperatures and thus, material viscosities. The agreement between the exponential approximation and data is slightly closer than for the vented bubble models, particularly as ${\bar{t}}_{b}$ approaches unity.

## 5. Discussion

### (a) The permeability problem

Figure 6 shows that the high temperature data deviate from all models at high values of ${\bar{t}}_{b}$. In particular, the divergence of the data from the vented bubble models as ${\bar{t}}_{b}$ approaches unity shows that those models are insufficient when the pores shrink to small sizes and the pore network closes. We explore this discrepancy by first acknowledging an implicit assumption of the vented bubble model formulation: that the gas permeability is sufficiently high that the gas pressure in the bubbles is always equal to the ambient pressure outside of the sample (and to the liquid pressure). We conjecture that the mismatch between model and data could be a consequence of a decrease in permeability during the evolution of the closing network of pores between the liquid droplets. As permeability decreases, outflow of gas is hindered, and the gas pressure can increase above the ambient, retarding the sintering process relative to the vented bubble model prediction. To test this theory, we can interrogate Darcy's law to extract the Darcy timescale λ_*D*_ when the gas is driven through the network by the capillary pressure in the pores, such that

5.1 $$\lambda_{D}=\frac{\mu_{g}L^{2}a}{k\Gamma },$$

where μ_*g*_ is the gas viscosity, *L* is the sample lengthscale (in our case the sample length itself; figure 2), and *k* is the gas permeability. We can estimate *k* for our initial system by using the universal scaling for impenetrable (hard) sphere packings proposed by Martys *et al.* [39], which was validated for glass sphere packings, sandstones and sintered materials [40]:

5.2 $$k=\frac{2(1-\phi +\phi_{c})}{s^{2}}{\left(\phi -\phi_{c}\right)}^{4.2},$$

where *s* is the specific surface area of the pores (i.e. the surface area of the pores normalized to the sample volume). This yields values of *k* for our initial system of approximately 10^−11^ m^2^ using values of 〈*a*_i_〉 estimated following the approach in §2f. It should be noted that Martys *et al.* [39] offer a second, very similar solution which is dependent on the *n*th moments of the particle size distribution; however, this is less easy to implement as a function of time as we are concerned with the evolution of pores and not particles. Using values appropriate for our initial system (*L*=2.98 mm and μ_*g*_=10^−5.5^ Pa s for argon gas), equation (5.1) predicts λ_*D*_≈10^−2.22^ s.

To assess how λ_*D*_ evolves with time, we need to know not just *k* and *a* for the initial system, but how these parameters evolve with time. The length *L* is extracted, as a function of time, from the image analysis described in §3 and is continuously recorded by the optical dilatometer. The radius of the pores as a function of the porosity is given by

**Figure 7.**
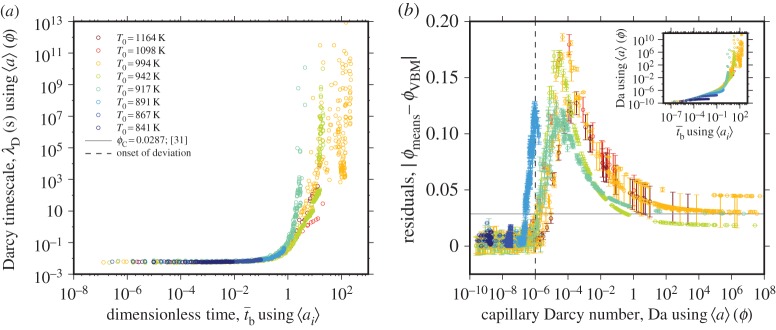
(*a*) Testing the proposition that the Darcy timescale λ_*D*_ (using *a*(*ϕ*) calculated from equation (5.3)) results in a permeability-inhibited sintering at high values of ${\bar{t}}_{b}$ (see §5a). (*b*) The residual between the measured porosity and the vented bubble model prediction as a function of the Capillary Darcy number Da (using 〈*a*〉(*ϕ*) calculated from equation (5.3)). The predicted critical porosity *ϕ*_*c*_ for polydisperse spheres [32] is marked by a horizontal line. *Inset*: the same residuals as in (*b*) as a function of the dimensional permeability calculated using equation (5.2) showing that during the permeability-decreasing sintering process, the first deviation from the model prediction occurs below *k*∼10^−12.6^−10^−12.2^. (Online version in colour.)

5.3 $$a=a_{i}{\left[\frac{\phi (1-\phi_{i})}{\phi_{i}(1-\phi )}\right]}^{1/3},$$

and we know that the surface area of spherical pores of radius *a* can be converted to a bulk specific surface area by *s*=3*ϕ*/*a* assuming, for these illustrative purposes, a monodisperse value of *a*. Equation (5.3) allows us to convert *ϕ*(*t*) (see §3) into *a*(*t*) and *s*(*t*). Now equation (5.1) can be assessed as a function of time, or more usefully, of ${\bar{t}}_{b}$ (figure 7*a*). We can see that the Darcy timescale is a strongly nonlinear function of time and goes toward infinite values at high values of ${\bar{t}}_{b}$. We propose that the increase of the Darcy timescale during sintering may produce a competition between the gas flow rate out of the sample and the sintering rate induced by the Laplace pressure. This competition is best seen when we define λ_*D*_/λ_*b*_ which yields a Capillary Darcy number

5.4 $$\text{Da}=\frac{\lambda_{D}}{\lambda_{b}}=\frac{\mu_{g}L^{2}}{\mu k},$$

when Da>1, the sintering timescale is shorter than the gas escape timescale.

If equation (5.4) represents the competition of timescales that is responsible for the discrepancy between our experimental data and the vented bubble model, we would expect that, as sintering progresses, the system should transition from Da<1 (i.e. sintering is unimpeded by permeable gas escape) to Da>1 (i.e. sintering is impeded by permeable gas escape) prior to attainment of volume equilibrium at *ϕ*_c_. In figure 7*b*, we show the residuals when we subtract the measured gas volume fraction *ϕ*_meas_ from that which is predicted by the vented bubble model *ϕ*_VBM_ as a function of the calculated Da. Here a residual of zero represents perfect agreement between *ϕ*_meas_ and *ϕ*_VBM_. At very low values of Da the residuals are approximately 0, suggesting that, when the permeability of the system is high, the model well predicts the sintering. However, at $\text{Da}≳10^{-6}$ the residuals increase, indicating that the vented bubble model underestimates *ϕ*. At Da∼10^−6^ the calculated permeabilities of the samples fall in the range $10^{-12.6}≲k≲10^{-12.2}\;{\text{m}}^{2}$. We suggest that these values represent the permeability below which the gas phase is not able to escape the sample unimpeded such that the free-venting assumption of the vented bubble model is violated. The peak residual occurs at Da∼10^−4^ after which the residuals decrease as the samples approach final porosity. Final gas volume fraction is associated with a residual of approximately 0.03, and Da in the range $10^{2}≲\text{Da}≲10^{8}$.

If decreasing sample permeability were responsible for the mismatch between data and models as the sintering process nears completion, we might expect the mismatch to be greatest for Da≈1. In fact, both the onset of the deviation between *ϕ*_meas_ and *ϕ*_VBM_, and the peak residual, occur while Da≪1 for which, as stated, Da is calculated for the bulk sample. Another alternative explanation for the mismatch arises from consideration that *a* is not monodisperse (§2f). The consequence is that isolation of pores may occur over a range of ${\bar{t}}_{b}$ such that the transition from permeable to impermeable is not a discrete event when assessed on the sample lengthscale [9]. This argument leads to the prediction that the metric of isolated gas volume fraction, *ϕ*_iso_, would be an increasing function of time at $10^{-6}≲\text{Da}≲10^{8}$ reaching stable values at high ${\bar{t}}_{b}$ for which *ϕ*_iso_=*ϕ*_c_. These propositions certainly require further investigation, which could be accomplished via *in situ* 4D experimental datasets.

**Figure 8.**
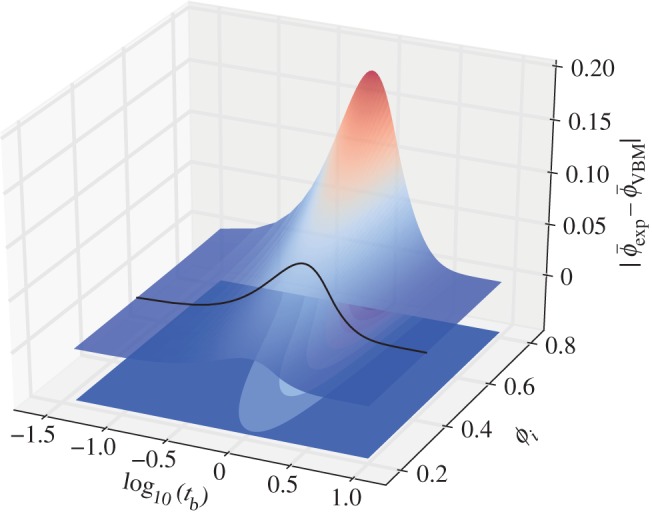
The difference between the exponential approximation (equation (2.19)) and the vented bubble model (equation (2.13)) showing that while at low values of *ϕ*_i_, there is reasonable agreement, this is not the case at high *ϕ*_i_. Our measured *ϕ*_i_ is marked as the black line on the curved surface. (Online version in colour.)

We have shown that, although the exponential approximation includes the erroneous implicit assumption that *a*≠*f*(*t*), it nonetheless provides the best fit to data of all the models that we present, consistent with its widespread adoption in the literature [7,8,19]. Given that the exponential approximation always overestimates *ϕ* compared with the vented bubble model (figure 8), while the vented bubble model tends to underestimate *ϕ* compared with the data at moderate and high Da, we speculate that the good performance of the exponential model might result from a fortuitous cancelling of errors. Figure 8 shows that the poorer performance of the exponential model might be expected if *ϕ*_i_ were larger than that tested here and in other studies.

### (b) The critical gas volume fraction and volume equilibrium

The critical value of gas volume fraction at which pores become completely isolated has been the subject of extensive investigation, and the percolation threshold for monodisperse systems of fully penetrable spheres has been found to be in the range 0.033<*ϕ*_c_<0.034 for any monodisperse system [14,15]. Investigation of the critical gas volume fraction for polydisperse fully penetrable spheres has suggested a small effect of polydispersivity, that results in a lower value of *ϕ*_c_=0.029 [32]. These studies use stochastic methods to randomly locate spheres in volumes such that the number density of the overlapping spheres controls the bulk solid volume fraction. In our system, however, we have a constant mass of liquid (equivalent to their solid volume fraction) and it is the volume of the whole system that controls the bulk liquid fraction. We find a critical gas volume fraction of *ϕ*_c_=0.03±0.008 for all temperatures investigated. This is in excellent agreement with these previous works [14,15]; however, the resolution of this value, manifest as the quoted uncertainty, is not sufficient to conclude whether our system of spheres is closer to the theoretical values of *ϕ*_c_ for the monodisperse [14,15] or the polydisperse [32] simulations. These results are summarized in table 2.

The *ϕ*_c_ discussed above is the equilibrium value, encapsulated by our model when we set *ϕ*_*f*_ to the empirically constrained *ϕ*_c_ (see §2e). However, there are processes that can modify *ϕ*_c_ from equilibrium by halting viscous flow prior to the completion of sintering. If sintering is coincident with crystallization of the liquid, or if sintering occurs during cooling through the glass transition, then sintering can stop prior to attainment of the equilibrium *ϕ*_c_. In a crystallizing system of sintering droplets, high crystal volume fractions can result in a local yield stress, which may be larger than the surface tension stress driving flow at the droplet surfaces, or can result in a jammed state in which bulk flow is altogether inhibited [41]. However, when liquid droplets are the size of those studied here, rigid crystals tend to form at their surface rather than in the droplet interiors [42]. A model treatment of the effect of this surface phenomenon on sintering rates remains enigmatic, particularly as the viscous flow driven by surface tension is also dominantly local to the surface.

Eberstein *et al.* [26] investigated systems of glass fragments mixed with rigid crystal particles and found that the empirically observed maximum linear shrinkage of cylindrical samples (manifest as the minimum final sample height *L*_*f*_) scales with a bulk crystal volume fraction *ϕ*_*x*_ such that $L_{f}\propto \phi_{x}^{3}$, which implies that the effect of crystallization on the final volume of the sample is a continuous function rather than an abrupt change when *ϕ*_*x*_ exceeds the crystal fraction above which the crystal suspension is jammed at the surface. Therefore, sintering can halt before the crystal-free *ϕ*_c_ is attained. Beyond this observation, a model which satisfactorily scales *ϕ*_*f*_ with *ϕ*_*x*_ is lacking and would be a valuable future contribution. Parametrization of the processes that affect the viscous sintering rate, including crystallization, glass composition, non-isothermal trajectories and droplet initial geometries, have been investigated in detail due to their application to ceramic and glass fabrication [8,25,43] or to natural systems in which sintering takes place, such as volcanic interiors [2,9]. However, further constraint of the parameters affecting *ϕ*_*f*_ is needed (as discussed above). The concept of volume equilibrium is therefore one that requires knowledge of the time- and temperature dependence of the material properties and we suggest that attainment of the equilibrium *ϕ*_c_ is reserved for a special class of metastable undercooled liquids which do not readily crystallize.

## 6. Conclusion

We show that viscous sintering can be modelled using a modified version of the original Mackenzie & Shuttleworth [6] theory. We test this and other models and discuss their limitations for real systems of liquid droplets. We find an equilibrium final gas volume fraction preserved in the samples that is in agreement with theoretical values for overlapping penetrable spheres. Finally, we introduce a conceptual framework for the incorporation of gas permeability into a sintering Darcy timescale for gas escape, and show that decreasing gas permeability may have an important impact on late-stage sintering rates. We propose that the most pressing unresolved complexities in the sintering of high-viscosity droplets, namely the low-permeability final phase of the sintering process identified here, could be explored by future high temperature *in situ* X-ray techniques.

## Supplementary Material

Supplementary Information document

## Supplementary Material

Processed data

## Data accessibility

Processed data are available in the electronic supplementary file and raw data are available upon request from the authors.
