# Supplementary material for: Sintering of viscous droplets under surface tension
Source: Proc Math Phys Eng Sci. 2016 Apr;472(2188):20150780. doi: 10.1098/rspa.2015.0780 (PMC4892276; doi:10.1098/rspa.2015.0780)
Supplement: Supplementary Information document [file rspa20150780supp1.pdf]

Sintering of viscous droplets under surface tension

Fabian B. Wadsworth[1,ᶠ], Jérémie Vasseur[1], Edward W. Llewellin[2], Jenny Schauroth[1], Katherine J. Dobson[1], Bettina Scheu[1], Donald B. Dingwell[1]

1 Department for Earth and Environmental Sciences, Ludwig Maximilian University, Theresienstr.41, 80333 Munich, Germany

2 Department of Earth Sciences, Durham University, Science Labs, Durham, DH1 3LE, United Kingdom.

[ᶠ]Corresponding author is Fabian Wadsworth: fabian.wadsworth@min.uni-muenchen.de

-- SUPPLEMENTARY FILE --

Deriving the vented bubble model from first principles (after Mackenzie & Shuttleworth (5) and Proussevitch & Sahagian (22))

Here we show how one can arrive at Eq. 2.13 (see §2.3) from first principles (see Figure 1C for geometry). Consider a spherical bubble of radius a centred in a spherical shell of melt with outside radius S (Figure 1C), where the melt has constant Newtonian viscosity μ and the interfacial tension is Γ. According to Eq. 7 in Proussevitch and Sahagian (22), the growth of the bubble under a pressure differential is given by

$$P_g - P_f = \frac{2\Gamma}{a} + 4\mu\left(\frac{1}{a} - \frac{a^2}{S^3}\right)\frac{da}{dt}, \qquad \text{Eq. S1}$$

where P_g and P_f are the pressure in the bubble and fluid respectively. If the gas in the bubble is able to escape unhindered (the "vented bubble" concept), then $P_g = P_f$ and we can rearrange for the bubble wall velocity

$$\frac{da}{dt} = -\frac{\Gamma S^3}{2\mu(S^3 - a^3)}. \qquad \text{Eq. S2}$$

The melt volume is constant over time so that

$$S^3 - a^3 = c\,, \qquad \text{Eq. S3}$$

and S and R are related to the gas volume fraction ϕ by

$$\phi = \frac{a^3}{S^3}\,, \qquad \text{Eq. S4}$$

hence we obtain a differential equation in a

$$\frac{da}{dt} = -\frac{\Gamma(a^3 + c)}{2\mu c}\,. \qquad \text{Eq. S5}$$

We can try to solve for $a(t)$ by separating variables

$$\int \frac{1}{a^3 + c}\,da = -\frac{\Gamma}{2\mu c}\int dt\,. \qquad \text{Eq. S6}$$

We can also solve Eq. S5 directly numerically, and we can calculate analytical solutions for the cases where $a^3 \gg c$ and $a^3 \ll c$, which respectively represent very large and very small ϕ. For $a^3 \gg c$ we have

$$\int \frac{1}{a^3}\,da = -\frac{\Gamma}{2\mu c}\int dt\,, \qquad \text{Eq. S7}$$

which gives

$$-\frac{1}{2a^2} + m = -\frac{\Gamma}{2\mu c}t\,. \qquad \text{Eq. S8}$$

Given the initial condition that $a = a_i$ when $t = 0$, we can evaluate m to find $a(t)$

$$a = \left(\frac{1}{{a_i}^2} + \frac{\Gamma}{\mu c}t\right)^{-\frac{1}{2}}, \qquad \text{Eq. S9}$$

i.e. the radius shrinks with $1/\sqrt{t}$. Similarly, for $a^3 \ll c$, we have

$$\frac{1}{c}\int da = -\frac{\Gamma}{2\mu c}\int dt\,, \qquad \text{Eq. S10}$$

which gives

$$a + m = -\frac{\Gamma}{2\mu}t\,. \qquad \text{Eq. S11}$$

With the same initial condition as before, we find

$$a = a_i - \frac{\Gamma}{2\mu} t \,, \qquad \text{Eq. S12}$$

i.e. the radius shrinks with t. We can now scale to $\phi(t)$ recasting Eq. S5 in terms of ϕ. Using the chain rule, we have

$$\frac{d\phi}{dt} = \frac{d\phi}{da}\frac{da}{dt} \,. \qquad \text{Eq. S13}$$

From Eq. S3 and S4 we have

$$\phi = \frac{a^3}{c + a^3} \,, \qquad \text{Eq. S14}$$

hence

$$\frac{d\phi}{da} = \frac{3\phi}{a}(1 - \phi) \,. \qquad \text{Eq. S15}$$

From Eq. S5 and S14, we have

$$\frac{da}{dt} = -\frac{\Gamma a^3}{2\mu c \phi} \,, \qquad \text{Eq. S16}$$

so, combining Eq. S13, S15 and S16, we have

$$\frac{d\phi}{dt} = -\frac{3\Gamma}{2\mu c}(1 - \phi)a^2 \,. \qquad \text{Eq. S17}$$

With further manipulation via Eq. S14, we can eliminate a^2 to give

$$\frac{d\phi}{dt} = -\frac{3\Gamma}{2\mu}\left(\frac{1 - \phi}{c}\right)^{\frac{1}{3}} \phi^{\frac{2}{3}} \,. \qquad \text{Eq. S18}$$

Noting that $a = a_i$ when $\phi = \phi_i$ and, again, using Eq. S14, we can eliminate c, expressing Eq. S18 in terms of observable starting parameters:

$$\frac{d\phi}{dt} = -\frac{3\Gamma}{2\mu a_i}\left(\frac{1 - \phi}{1 - \phi_0}\right)^{\frac{1}{3}} \phi^{\frac{2}{3}} {\phi_0}^{\frac{1}{3}} \,. \qquad \text{Eq. S19}$$

If we now normalize ϕ by the starting porosity ϕ_i such that

$$\bar{\phi} = \frac{\phi}{\phi_i} \,, \qquad \text{Eq. S20}$$

Eq. S19 becomes

$$\frac{d\bar{\phi}}{dt} = -\frac{3\Gamma}{2\mu a_i}\left(\frac{1-\phi_i\bar{\phi}}{1-\phi_i}\right)^{\frac{1}{3}}\bar{\phi}^{\frac{2}{3}}. \qquad \text{Eq. S21}$$

Note that $\mu a_i/\Gamma = \bar{t_b}$ defines a characteristic timescale which we can use to non-dimensionalize time:

$$\bar{t_b} = \frac{\Gamma}{\mu a_i}t\,, \qquad \text{Eq. S22}$$

giving us a dimensionless form of Eq. S21

$$\frac{d\bar{\phi}}{d\bar{t_b}} = -\frac{3}{2}\left(\frac{1-\phi_i\bar{\phi}}{1-\phi_i}\right)^{\frac{1}{3}}\bar{\phi}^{\frac{2}{3}}. \qquad \text{Eq. S23}$$

As before, this can be solved numerically, and has the advantage of non-dimensionality. In the main text of this work, we present a solution for small ϕ_i (Eq. 2.15). Additionally, we can solve for the behaviour at small $\bar{t_b}$ (when $\phi \approx \phi_i$; hence, $\bar{\phi} \approx 1$):

$$\frac{d\bar{\phi}}{d\bar{t_b}} = -\frac{3}{2}. \qquad \text{Eq. S24}$$

Integrating, we obtain $\bar{\phi}(\bar{t_b})$:

$$\bar{\phi} = 1 - \frac{3}{2}\bar{t_b}\,, \qquad \text{Eq. S25}$$

or, dimensionally

$$\phi = \phi_i\left(1 - \frac{3\Gamma}{2\mu a_i}t\right) \qquad \text{Eq. S26}$$

which shows that ϕ decreases linearly with time at the onset of sintering, congruous with our experimental results.

Non-isothermal forms of Eq. S23 and S26 can be achieved using the same approach as in the main text of our work (via Eq. 2.6).

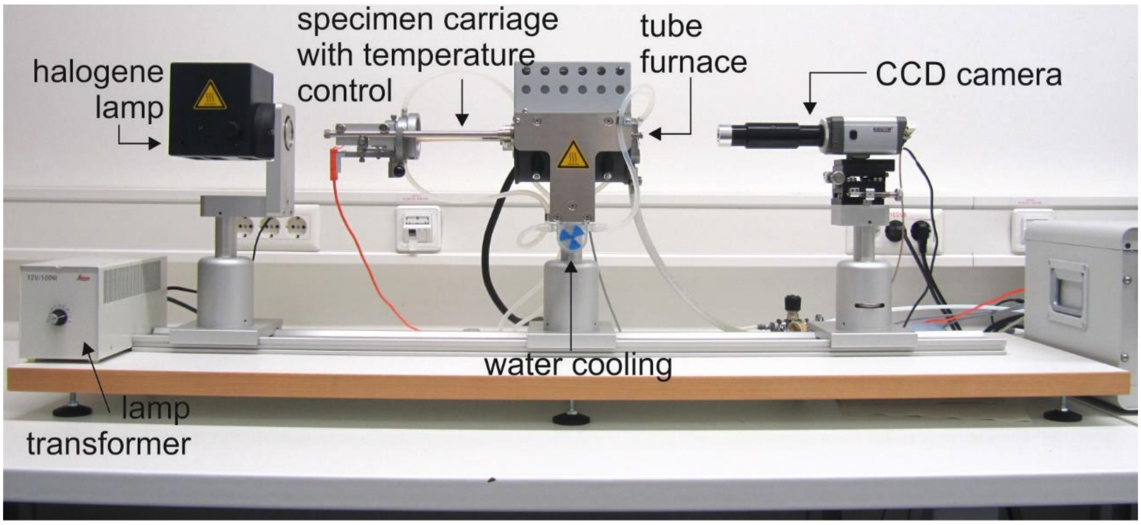

Supplementary Figure 1. A schematic of the optical dilatometer equipment (see main text §3 for details).

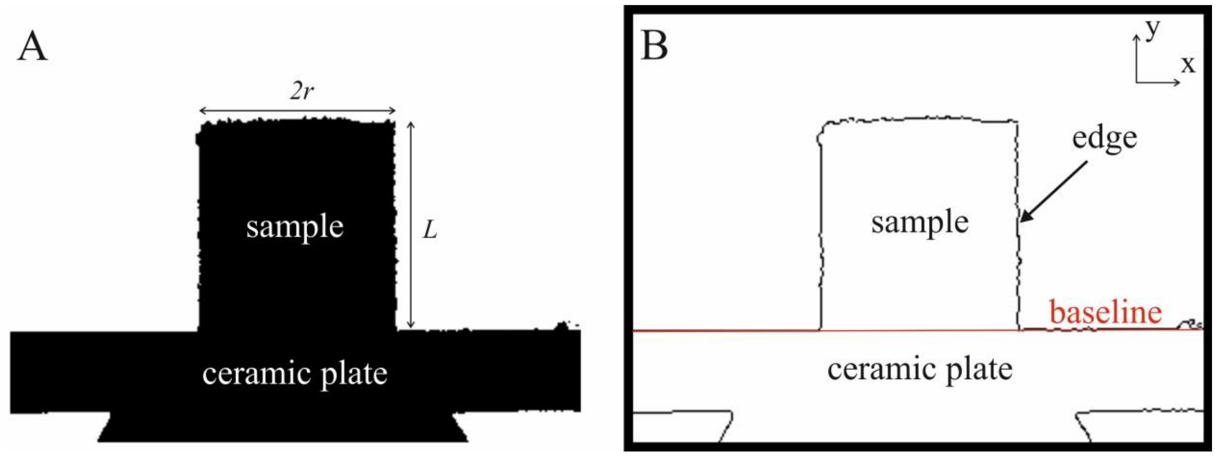

Supplementary Figure 2. (A) An example binary sample image. **(B)** The same image after the Canny edge detection is applied discretizing the sample edge position and the finding of the baseline.

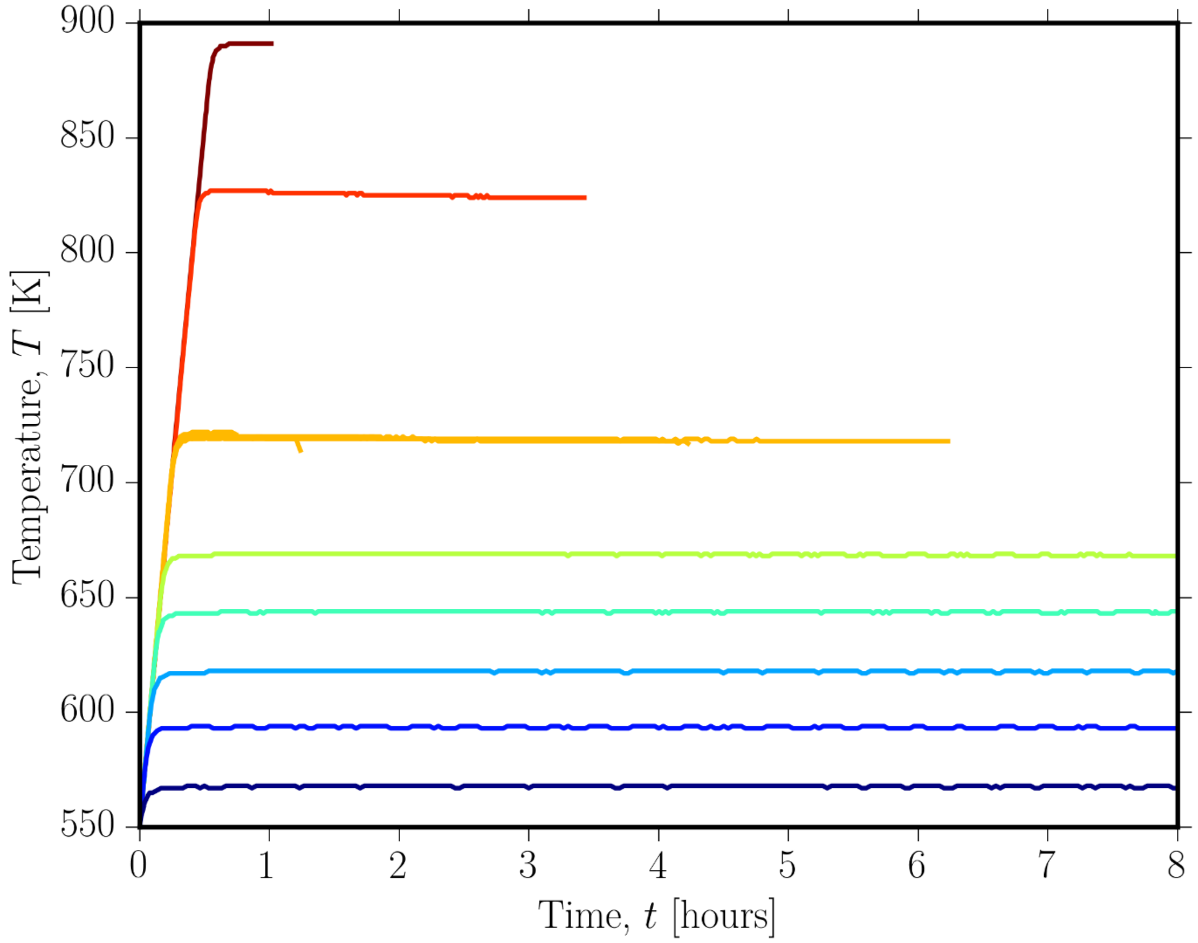

Supplementary Figure 3. The evolution of sample temperature as a function of time during measurement of sample geometry. The thermocouple is within 1.5 mm of the base of the sample. The heating rate prior to the isotherm is 10 $K.min^{-1}$ (see main text §3 for details).

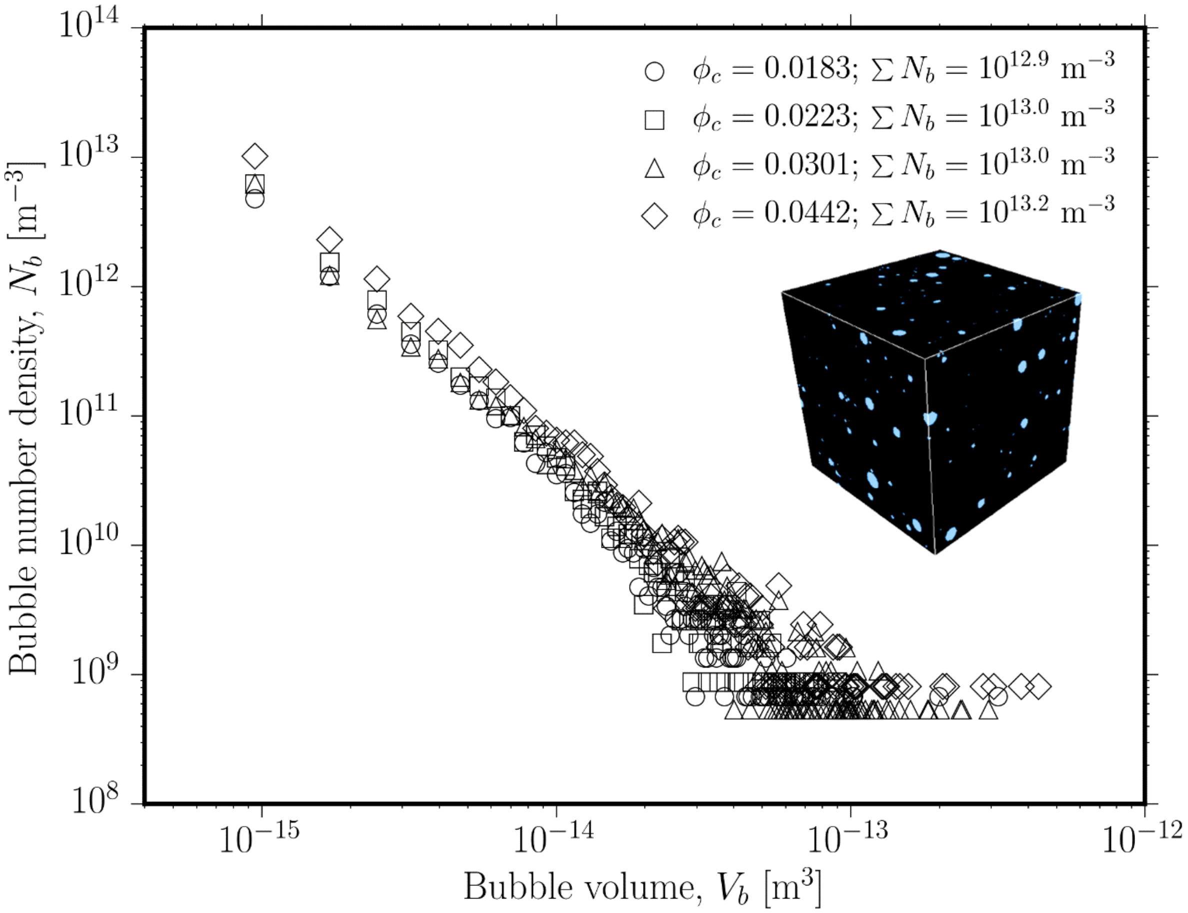

Supplementary Figure 4. The bubble volumes V_b as a function of bubble number density N_b preserved in experiments which were quenched at the end of the sintering process. The values of ϕ_c are labelled (see text). *Inset* an example of the segmented pores on a box-edge plot of the sub volume (box edge of ~100 µm) analysed by x-ray computed tomography.
